# Anterior Approach for Fixation of Acetabular Fractures Using Anatomically Designed Plates: Maintenance of Reduction and Functional Outcomes at a Minimum Five-Year Follow-Up

**DOI:** 10.7759/cureus.73079

**Published:** 2024-11-05

**Authors:** Tristan R Fraser, Mohamed A Khalefa, Tim Chesser, Anthony J Ward, Mehool Acharya

**Affiliations:** 1 Orthopaedic Surgery, North Bristol NHS Trust, Bristol, GBR; 2 Orthopaedics, Cairo University, Cairo, EGY; 3 Orthopaedics, The Royal Orthopaedic Hospital, Birmingham, GBR

**Keywords:** acetabular fractures, anterior intrapelvic approach, patient-related outcome measures, pelvic acetabular trauma, quadrilateral plate, specialized surgical instruments

## Abstract

Background

Acetabular fractures with quadrilateral plate involvement have been shown to have a high rate of complications. Anatomic suprapectineal plating systems have been developed to manage these injuries with good short-term outcomes. However, long-term maintenance of anatomical reduction and functional outcomes has yet to be established. The aim of this study is to maintain reduction and functional outcomes at a minimum of five years of follow-up.

Materials and methods

This is a retrospective cohort study from a prospective database examining patients aged over 16 years following fixation of acetabular fractures with quadrilateral plate involvement at a trauma center in the United Kingdom. All patients had acetabular fracture fixation with an anatomically designed suprapectineal plate. Patients were admitted from March 2014 to January 2017. Primary outcomes included objective radiological outcomes such as reduction quality, maintenance of reduction, and subjective patient-related outcome measures (PROMs) using the Oxford Hip Score (OHS) and EuroQol EQ5D Score at a minimum of five years post-operatively. Secondary outcomes recorded included metalwork failure and complications such as reoperation, neurological deficit, and mortality.

Results

16 patients met our eligibility criteria in this cohort. Post-operative mean OHS at a minimum of five years was 40.5 (SD=11.9), with a median score of 45. Post-operative mean EuroQol EQ-5D scores at a minimum of 5 years were 0.83 (SD=0.25).

Radiographic outcomes were assessed with AP and Judet plain radiographs at a minimum of five years follow-up. Preoperatively, 56.3% (9) showed evidence of dome comminution, with 18.8% (3) demonstrating dome impaction. 93.7% (15) had quadrilateral plate involvement. 12.5% (2) showed evidence of femoral head injury. The rate of conversion to total hip replacement was 6.25% (1) at 15 months post-injury.

Conclusions

Maintenance of reduction and functional and patient-reported outcomes of patients who underwent open reduction and internal fixation of an acetabular fracture using anatomically contoured suprapectineal plates have satisfactory radiological and functional outcomes at five-year follow-up.

## Introduction

Pelvic and acetabular fractures comprise 2-8% of the United Kingdom’s annual fracture burden, with an incidence of three per 100,000 per annum [1]. Within this heterogenous group of injuries, acetabular fractures have been shown to have a high rate of complications, with 20-25% of patients demonstrating poor outcomes in the medium term [2]. The gold standard for displaced fractures not suitable for non-operative management is the open anatomical reduction and internal fixation (ORIF) performed in dedicated units by specialist surgeons [3].

Factors influencing the outcome of these injuries include fracture pattern, delay to surgery, age, chondral damage (femoral and acetabular), dislocation at the time of injury, neurovascular compromise, and pre-existing co-morbidities [4,5]. Radiographic features that unfavourably influence outcomes include severe acetabular impaction and marked acetabular displacement [6], with the anatomical restoration of the acetabular dome being a crucial factor in clinical outcomes [7].

The surgical approach for acetabular injuries is dictated by which specific portions of the osseous pelvis require access, the type of fixation, and the surgeon’s training and expertise [8,9]. Approaches are divided according to whether the anterior or posterior columns need to be accessed. Anterior approaches include the ilioinguinal, anterior intrapelvic (AIP/Stoppa), Iliofemoral, pararectus, and modifications of these approaches. The posterior column is typically accessed using a Kocher-Langenbeck approach.

Patients with quadrilateral plate involvement are a challenging group to manage, and anatomical reduction and stable fixation remain essential in order to achieve a satisfactory outcome [10]. A variety of instruments and implants have been developed to help achieve the anatomical reduction of displaced quadrilateral plate fractures, such as AIP approach-specific instruments and retractors, as well as anatomic suprapectineal quadrilateral surface plating systems. The plate is designed to allow it to buttress the quadrilateral surface while allowing for screw fixation along both the posterior column and the pelvic brim. 

A case series performed at Southmead Hospital Major Trauma Centre showed that with this system, surgeons were able to achieve anatomical reduction in 82% of patients, relatively low complication rates, and satisfactory functional outcomes in one year [11].

This study aims to assess the maintenance of reduction and functional outcomes at a minimum five-year follow-up.

## Materials and methods

**Figure 1:**
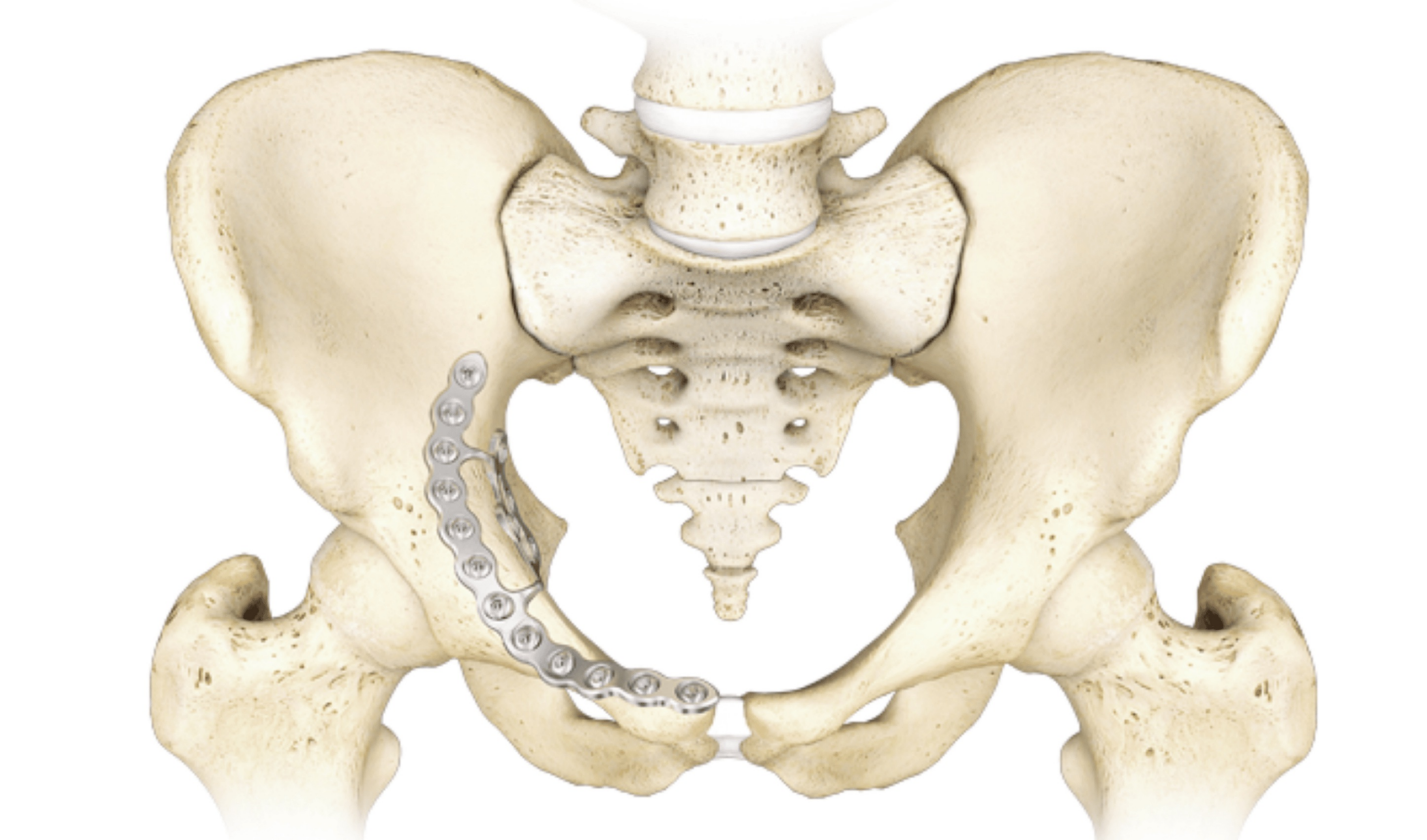
Stryker PRO anatomically-designed suprapectineal plate Reprinted with permission from ^©^Stryker Corporation. All rights reserved.

A retrospective cohort study examining patients aged over 16 years who underwent open reduction and internal fixation of acetabular fractures with quadrilateral plate involvement at a tertiary trauma center and specialist pelvic reconstruction unit in the United Kingdom was performed. All patients had an anterior approach for acetabular fracture fixation with an anatomically designed suprapectineal plate (PRO-Pelvis and Acetabulum System, Stryker, Kalamazoo, MI) (Figure 1) [12]. Patients were admitted from March 2014 to January 2017. The following data parameters were collected from medical records: age on admission, sex, mechanism of injury, and delay to surgery. Exclusion criteria included the need for an additional posterior approach to facilitate fracture reduction and fixation.

The standard pelvic and acetabular fracture management (of the department) pre-operative workup, surgical approach, and post-operative care were followed, with the only difference in management in this series being the implementation of the acetabular fixation system. Patients underwent an anterior intrapelvic approach (AIP) with the extension of the incision using lateral and/or medial windows based on the ability to achieve anatomical reduction. After reduction was achieved using specialist pelvic clamps, the reduction was held with interfragmentary screws or Kirschner wires. An appropriately-sized pre-contoured anatomical pectineal plate was secured with screws placed posteriorly and anteriorly.

Post-operatively, patients underwent weight-bearing restrictions: four weeks of touch-weight bearing followed by four weeks of protected weight-bearing before returning to full weight-bearing following review in a dedicated pelvic clinic with post-operative radiographs at eight weeks post-operatively. Chemical venous thromboembolism prophylaxis was given for four weeks post-operatively.

Acetabular fracture pattern according to the Letournel classification based on plain pelvic and acetabular radiographs (anteroposterior, iliac oblique, and obturator oblique Judet views) as well as computed tomography (CT) scans were analyzed and recorded pre-operatively. Primary outcomes included objective radiological outcomes and subjective patient-related outcome measures (PROMs).

Radiological outcomes such as reduction quality according to Matta, maintenance of reduction, quadrilateral plate involvement, quadrilateral plate reduction, dome comminution, dome impaction, and femoral head injury were recorded from independent analysis of anteroposterior and Judet radiographs by TF (a resident doctor) and MA (a consultant pelvic surgeon) at a minimum of five-years post-operatively. Any disagreement in the analysis was discussed with TC (a consultant pelvic surgeon) in order to reach a consensus. Subjective patient-related outcome measures (PROMs) using the OHS and EuroQol EQ5D Score at a minimum of five years post-operatively were recorded.

Complications according to the Clavien-Dindo classification of post-operative complications, including reoperation, neurological deficit, venous thrombo-embolism, and mortality, were collected from medical records. Patients from the original study cohort were contacted via telephone to ask for their consent to participate in this study [11]. Patient-related outcome measures were taken over the telephone and patients were asked to attend the Southmead Hospital radiology department for repeat plain radiographs of their pelvis (anteroposterior and Judet views). Patients who did not answer the telephone were attempted to be contacted twice further. If they could still not be contacted following this, they were excluded from the study.

## Results

Patient demographics

16 patients met our eligibility criteria in this cohort. There were 14 male and two female patients with a mean age of 51 years at the time of injury (range 27-70 years, median 55 years).

Patterns of injury and treatment

The time from admission to operation was 3.82 days (range 1-9 days). Letournel fracture patterns included associated both columns (7), anterior column posterior hemi-transverse (6), transverse posterior wall (2), and anterior column (1). Anatomical reduction, based on analysis of post-operative imaging, was achieved in 94% (15) of the patients.

The most common mechanism of injury was cycling accidents, which attributed to 37.5% (6) of cases (Table 1).

**Table 1:** Mechanism of injury

Mechanism	Number (%)
Cyclist	6 (37.5%)
Fall greater than 1 meter	5 (31.2%)
Road traffic accident, pedestrian hit	2 (12.5%)
Fall, less than 1 meter	1 (6.2%)
Others	2 (12.5%)

Maintenance of reduction

Initial post-operative radiographic outcomes showed an anatomical reduction in 81.2% (13) and an imperfect reduction in 18.8% (3). There were no cases of poor reduction.

Radiographic outcomes

Radiographical evaluation for quality of reduction was undertaken, according to Matta [13]. The criteria were: excellent 4 mm or less, good 5 to 10 mm, fair 10 to 20 mm, and poor more than 20 mm. Analysis of post-operative imaging showed excellent outcomes in 62.5% (10), good outcomes in 31.25% (5), and poor outcomes in 6.25% (1 patient who underwent total hip arthroplasty 15 months post-injury).

**Figure 2:**
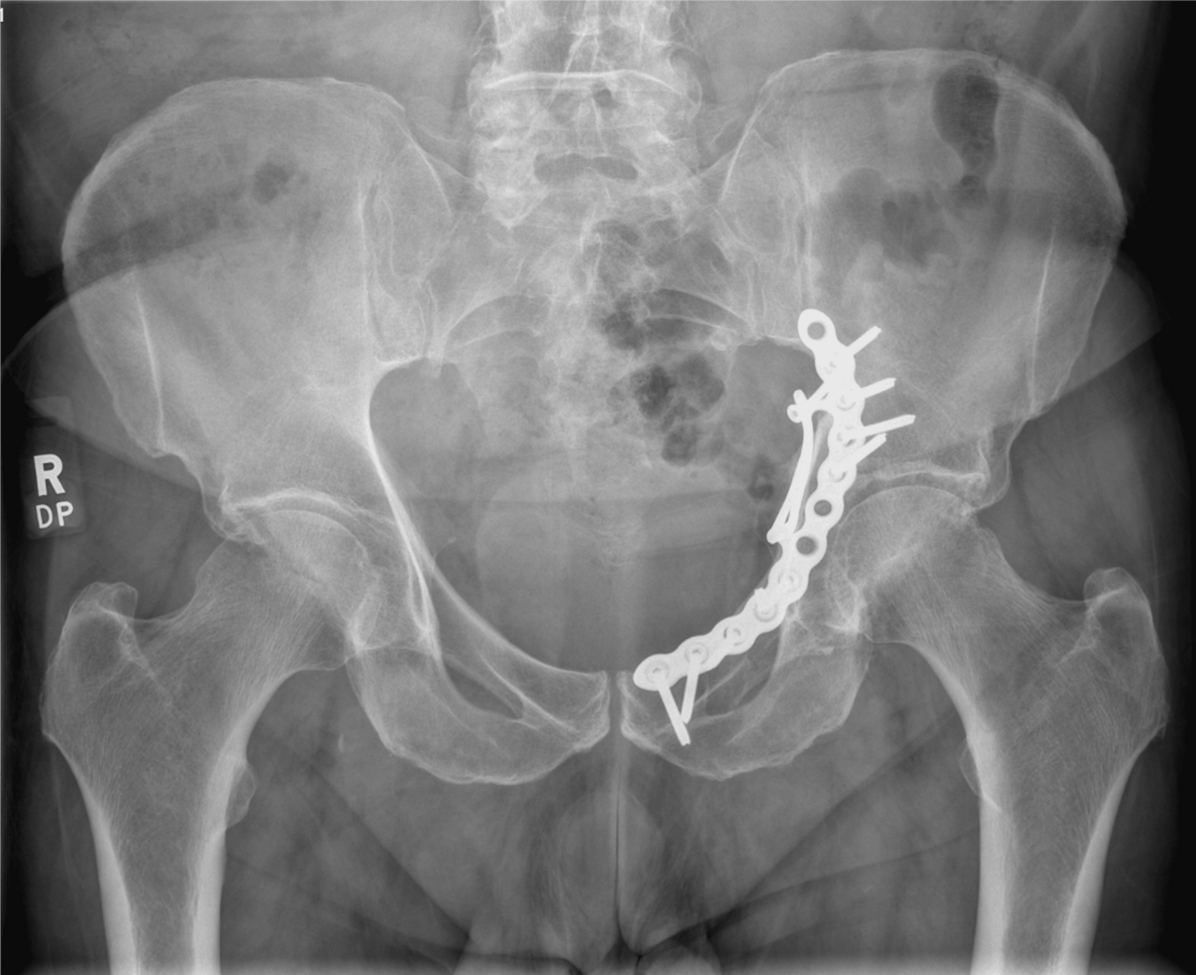
Anteroposterior pelvic radiograph in a patient, five years following fixation of a displaced acetabular fracture using an anatomical suprapectineal plate

**Figure 3:**
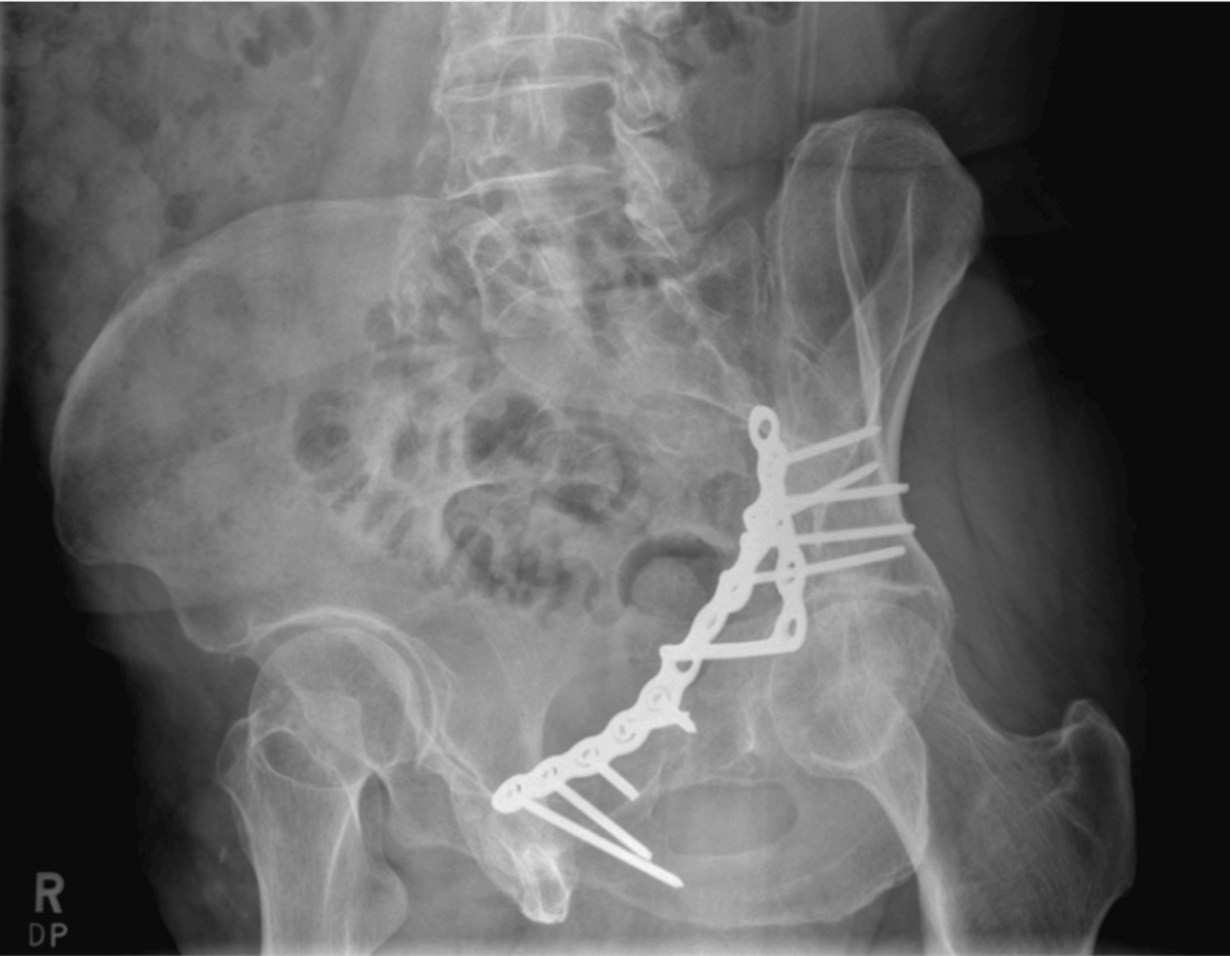
Obturator oblique radiograph of the left hip in a patient, five years following fixation of a displaced acetabular fracture using an anatomical suprapectineal plate

**Figure 4:**
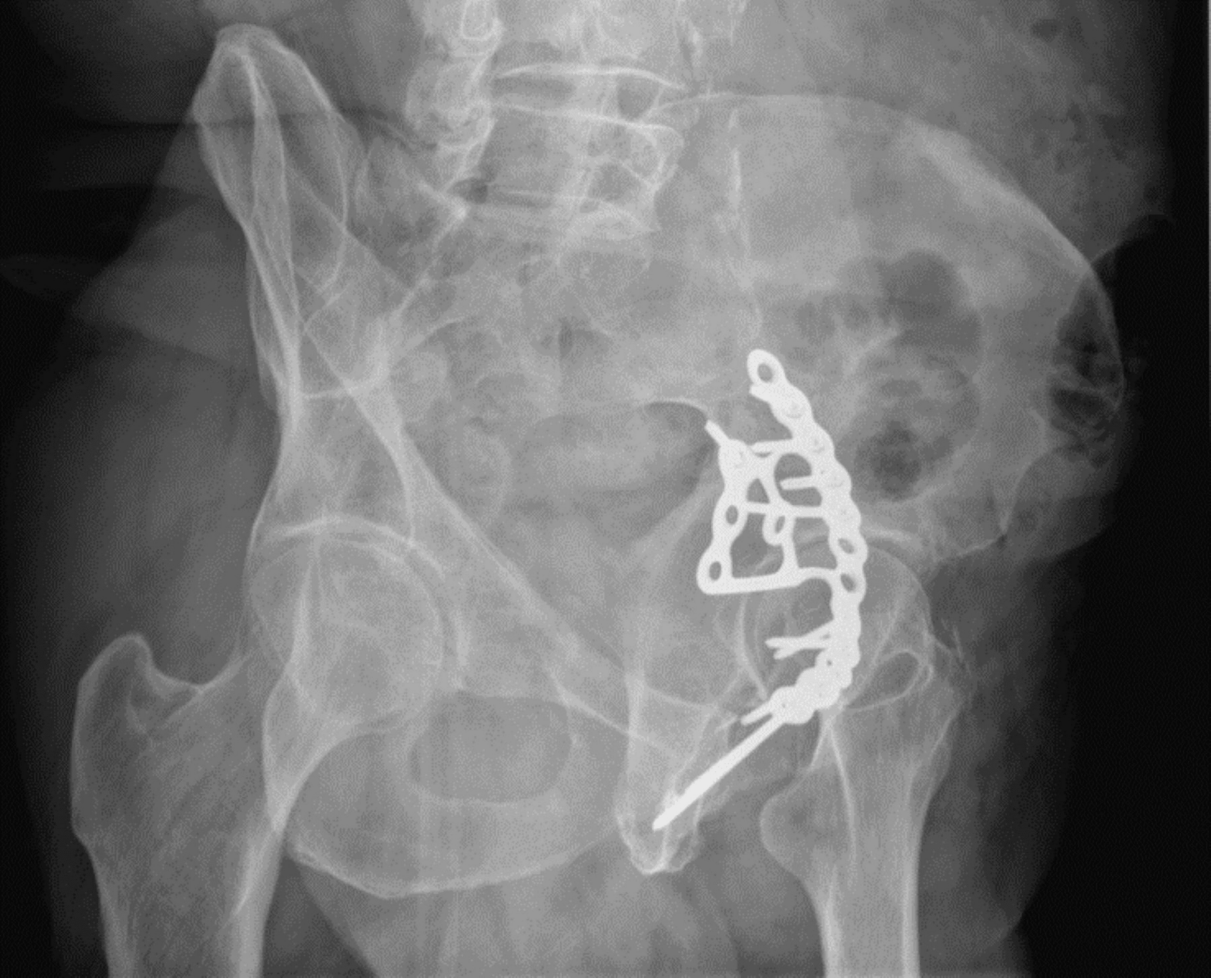
Iliac oblique radiograph of the left hip in a patient, five years following fixation of a displaced acetabular fracture using an anatomical suprapectineal plate

Radiographic outcomes were assessed with AP, iliac oblique, and obturator oblique Judet view plain radiographs, at a minimum of five years follow-up (Figure 2, Figure 3, Figure 4). Quadrilateral plate reduction was maintained in 93.7% (15) of the patients. 56.3% (9) showed some evidence of dome comminution, and 18.8% (3) demonstrated dome impaction. 83.7% (13) showed evidence of quadrilateral plate involvement. 12.5% (2) showed evidence of femoral head injury. The rate of re-operation and conversion to total hip replacement was 6.25% (1) at 15 months post-injury.

Functional outcomes

Outcomes were assessed at one year and five years post-operatively. Post-operative mean Oxford Hip Score (OHS) at a minimum of five years was 40.5 (SD=11.9), with a median score of 45. The post-operative mean EuroQol EQ5D score at a minimum five-year follow-up was 0.83 (SD = 0.25). Comparison of the Oxford Hip Score (OHS) and EQ5D scores with outcomes from a minimum one-year post-operative follow-up showed no significant changes at five years (OHS, p=0.27/EQ5D, p=0.128). 

Complications and mortality

Per the Clavien-Dindo classification for post-operative complication severity, one patient experienced a Grade II complication, presenting as deep venous thrombosis requiring anticoagulation. Two other patients had Grade III complications necessitating re-operation: one developed post-traumatic arthritis requiring hip replacement and, subsequently, a deep surgical site infection, which led to multiple debridements and implant removal at 10 months post-fixation.

## Discussion

Anatomical reduction of acetabular fractures is key to preventing conversion to arthroplasty [14]. While there was a single case of conversion to total hip arthroplasty (THA) within our cohort, this occurred early. We would suggest an OHS of <38 is an indicator of early conversion to THA. Otherwise, there was no significant difference in OHS between early follow-up and follow-up at a minimum of five years following index surgery.

The anterior intrapelvic (modified Stoppa) and ilioinguinal approaches have both been used to facilitate reduction of anterior acetabular fractures. Rates of anatomical reduction using various anterior approach techniques have been demonstrated in the literature. A systematic review [15] investigating rates of anatomical reduction, functional outcomes, complications and operative time demonstrated that whilst there is no significant difference in functional outcomes, the Anterior Intrapelvic approach demonstrated significantly superior rates of anatomical reduction, a significant reduction in complication rates and shorter operative time [16-19].

An observational case series (n=30) examining the functional outcomes (using the Merle d’Aubigné score) and radiological outcomes (according to Matta) of the Stryker PRO system at one-year follow-up was performed by Gras et al. Functional outcomes demonstrated a Merle d’Aubigne score of excellent in 17%, good in 37%, fair in 33% and poor in 13%. Matta grading was excellent in 50%, good in 25%, fair in 11% and poor in 14% [20].

Conventional plating systems are placed on the superior surface of the pelvic brim, with medial support created either with use of longer screws across the quadrilateral plate or by buttressing the quadrilateral plate from the suprapectineal area downwards [21]. Implants used for this purpose included using a standard pelvic plate as a spring plate, T-plates, H-plates or 1/3 semitubular plates. Studies investigating rates of fracture reduction using the modified Stoppa approach alongside conventional plating systems have shown rates of anatomical fracture reduction varying between 36.3 and 82.5% [18,22]. 

A retrospective analysis performed by Shazar et al. analysed radiographic outcomes in 225 patients who underwent open reduction and internal fixation of an acetabular fracture via either an AIP (n=103) or ilioinguinal approach (n=122) [18]. Anatomical reduction in the AIP cohort was achieved in 85 patients (82.5%) however no further follow-up and no patient-related outcome measures were provided.

A retrospective cohort study (n=11) performed by Sahin et al. analysed outcomes following treatment of acetabulum anterior wall fractures using open reduction and internal fixation using a modified Stoppa (AIP) approach [22]. Both radiographic outcomes and patient-related outcome measures using the modified Merle d'Aubigné score were recorded. Anatomical reduction was achieved in four patients (36.3%) and patients were followed up until two years post-operatively.

A limitation of this paper is the high proportion of patients lost to follow-up. Of the 33 patients in the original study cohort 12 were lost to follow-up, four had died at time of follow-up and one patient had moved abroad.

Acetabular fracture management with quadrilateral plate involvement require anatomical reduction to ensure adequate functional outcomes [3, 7]. This study demonstrates that the Stryker PRO system can help to achieve and maintain anatomical reduction at medium term follow up. The functional outcome in this group of patients remains satisfactory and is maintained at five years following index surgery, however it must be noted that whilst short and medium-term outcomes are promising, there is at present no long-term data on the outcomes of these implants. Use and implementation of any new products need to be monitored to ensure that results are comparable to the standard of care.

## Conclusions

This study demonstrates that open reduction and internal fixation of acetabular fractures in a specialist pelvic and acetabular reconstruction unit using anatomically-contoured pectineal plates maintains anatomical reduction at a minimum of five years follow-up in 94%. A poor Oxford Hip Score is an indicator of early conversion to total hip arthroplasty, however we have found that in patients with a satisfactory Oxford Hip Score at short-term follow-up have their function maintained at minimum five years follow-up. This method of fixation has an acceptable rate of maintenance of patient-reported outcome measures, maintenance of anatomical reduction, and a low rate of long-term complications. 

## References

[1] Laird A, Keating JF (2005). Acetabular fractures: a 16-year prospective epidemiological study. J Bone Joint Surg Br.

[2] Giannoudis PV, Bircher M, Pohlemann T (2007). Advances in pelvic and acetabular surgery. Injury.

[3] Briffa N, Pearce R, Hill AM, Bircher M (2011). Outcomes of acetabular fracture fixation with ten years' follow-up. J Bone Joint Surg Br.

[4] Giannoudis PV, Grotz MR, Papakostidis C, Dinopoulos H (2005). Operative treatment of displaced fractures of the acetabulum. A meta-analysis. J Bone Joint Surg Br.

[5] Murphy D, Kaliszer M, Rice J, McElwain JP (2003). Outcome after acetabular fracture. Prognostic factors and their inter-relationships. Injury.

[6] Mears DC, Velyvis JH, Chang CP (2003). Displaced acetabular fractures managed operatively: indicators of outcome. Clin Orthop Relat Res.

[7] Matta JM (1996). Fractures of the acetabulum: accuracy of reduction and clinical results in patients managed operatively within three weeks after the injury. J Bone Joint Surg Am.

[8] Cheng EY, Bastian JD (2019). Selecting surgical approaches for treatment of acetabular fractures. JBJS Essent Surg Tech.

[9] Chesser TJ, Eardley W, Mattin A, Lindh AM, Acharya M, Ward AJ (2015). The modified ilioinguinal and anterior intrapelvic approaches for acetabular fracture fixation: indications, quality of reduction, and early outcome. J Orthop Trauma.

[10] Ardiansyah A, Dilogo IH, Gunawan B, Oesman I, Herlambang D (2024). Functional, radiological, and quality of life outcome of unstable acetabular fracture with quadrilateral plate involvement at a tertiary care center in Jakarta, Indonesia. Eur J Orthop Surg Traumatol.

[11] Khalefa MA, El Nahal WA, Abdel Karim M, Abdel-Kader KF, Chesser TJ, Ward AJ, Acharya M (2022). Anterior approach for fixation of acetabular fractures using anatomically designed plates: accuracy of reduction and early functional outcomes with a minimum of 1-year follow-up. J Orthop Trauma.

[12] (2023). Stryker PRO - Pelvis Reduction and Osteosynthesis Plating System Pelvis and Acetabulum System Operative Technique. PRO-ST-1 Rev 3, 06-2017.. https://www.stryker.com/gr/en/trauma-and-extremities/products/pro-pelvis/index-eu.html.

[13] Matta JM, Tornetta P (1996). Internal fixation of unstable pelvic ring injuries. Clin Orthop Relat Res.

[14] Kiran M, Frostick R, Elnahal W, Spence D, Acharya M, Ward A, Chesser TJ (2023). The fate of acetabular fracture fixation at 10 years: rates of conversion to arthroplasty. Hip Int.

[15] Meena S, Sharma PK, Mittal S, Sharma J, Chowdhury B (2017). Modified stoppa approach versus ilioinguinal approach for anterior acetabular fractures; a systematic review and meta-analysis. Bull Emerg Trauma.

[16] Hammad AS, El-Khadrawe TA (2015). Accuracy of reduction and early clinical outcome in acetabular fractures treated by the standard ilio-inguinal versus the Stoppa/iliac approaches. Injury.

[17] Elmadağ M, Güzel Y, Acar MA, Uzer G, Arazi M (2014). The Stoppa approach versus the ilioinguinal approach for anterior acetabular fractures: a case control study assessing blood loss complications and function outcomes. Orthop Traumatol Surg Res.

[18] Shazar N, Eshed I, Ackshota N, Hershkovich O, Khazanov A, Herman A (2014). Comparison of acetabular fracture reduction quality by the ilioinguinal or the anterior intrapelvic (modified Rives-Stoppa) surgical approaches. J Orthop Trauma.

[19] Ma K, Luan F, Wang X (2013). Randomized, controlled trial of the modified Stoppa versus the ilioinguinal approach for acetabular fractures. Orthopedics.

[20] Gras F, Marintschev I, Grossterlinden L (2017). The anterior intrapelvic approach for acetabular fractures using approach-specific instruments and an anatomical-preshaped 3-dimensional suprapectineal plate. J Orthop Trauma.

[21] Ortega-Briones A, Smith S, Rickman M (2017). Acetabular fractures in the elderly: midterm outcomes of column stabilisation and primary arthroplasty. Biomed Res Int.

[22] Sahin I, Can F, Gultac E, Kilinc RM, Aydogan N, Kilinc CY (2023). Treatment of Acetabulum Anterior Wall Fractures Using the Modified Stoppa Approach. Cureus.

